# Adenosine stimulates the basolateral 50 pS K^+^ channel in renal proximal tubule via adenosine-A1 receptor

**DOI:** 10.3389/fphys.2023.1242975

**Published:** 2023-08-28

**Authors:** Hao Liu, Qi Sun, Zheng Ding, Wensen Shi, Wen-Hui Wang, Chengbiao Zhang

**Affiliations:** ^1^ Department of Physiology, Xuzhou Medical University, Xuzhou, China; ^2^ Department of Pharmacology, New York Medical College, Valhalla, NY, United States

**Keywords:** Kir4.2, Kir5.1, Na^+^ transport, NHE_3_, proximal tubule

## Abstract

**Background:** The basolateral potassium channels play an important role in maintaining the membrane transport in the renal proximal tubules (PT) and adenosine receptors have been shown to regulate the trans-epithelial Na^+^ absorption in the PT. The aim of the present study is to explore whether adenosine also regulates the basolateral K^+^ channel of the PT and to determine the adenosine receptor type and the signaling pathway which mediates the effect of adenosine on the K^+^ channel.

**Methods:** We have used the single channel recording to examine the basolateral K^+^ channel activity in the proximal tubules of the mouse kidney. All experiments were performed in cell-attached patches.

**Results:** Single channel recording has detected a 50 pS inwardly-rectifying K^+^ channel with high channel open probability and this 50 pS K^+^ channel is a predominant type K^+^ channel in the basolateral membrane of the mouse PT. Adding adenosine increased 50 pS K^+^ channel activity in cell-attached patches, defined by NP_o_ (a product of channel Numbers and Open Probability). The adenosine-induced stimulation of the 50 pS K^+^ channel was absent in the PT pretreated with DPCPX, a selective inhibitor of adenosine A1 receptor. In contrast, adenosine was still able to stimulate the 50 pS K^+^ channel in the PT pretreated with CP-66713, a selective adenosine A2 receptor antagonist. This suggests that the stimulatory effect of adenosine on the 50 pS K^+^ channel of the PT was mediated by adenosine-A1 receptor. Moreover, the effect of adenosine on the 50 pS K^+^ channel was blocked in the PT pretreated with U-73122 or Calphostin C, suggesting that adenosine-induced stimulation of the 50 pS K^+^ channels of the PT was due to the activation of phospholipase C (PLC) and protein kinase C (PKC) pathway. In contrast, the inhibition of phospholipase A2 (PLA2) with AACOCF3 or inhibition of protein kinase A (PKA) with H8 failed to block the adenosine-induced stimulation of the 50 pS K^+^ channel of the PT.

**Conclusion:** We conclude that adenosine activates the 50 pS K^+^ channels in the basolateral membrane of PT via adenosine-A1 receptor. Furthermore, the effect of adenosine on the 50 pS K^+^ channel is mediated by PLC-PKC signaling pathway.

## Introduction

The basolateral K^+^ channels in the PT play a role in the regulation of membrane transport in the PT (Wang et al., 1997). This notion is supported by the previous report that the deletion of inwardly-rectifying K^+^ channel 4.2 (Kir4.2), a major Kir channel in the PT, compromised the trans-epithelial bicarbonate reabsorption in the PT (Bignon et al., 2020). It is well established that Kir4.2 interacts with Kir5.1 to form a basolateral Kir4.2/Kir5.1 heterotetramer which is the major type of basolateral K^+^ channel and determines the membrane potential of the mouse PT (Tucker et al., 2000; Bignon et al., 2020; Lin et al., 2022). However, the conductance and biophysical properties of this Kir4.2/Kir5.1 heterotetramer in the native PT are largely unknown due to the technical difficulty to patch the basolateral membrane of the mouse PT. In this regard, several previous studies performed in the rabbit PT have identified a 50–60 pS inwardly-rectifying K^+^ channel, which is a main type of the basolateral K^+^ channel in the rabbit PT (Tsuchiya et al., 1992; Beck et al., 1993; Noulin et al., 1999). This suggests the possibility that Kir4.2/Kir5.1 heterotetramer in the mouse PT may be a 50–60 pS inwardly-rectifying K^+^ channel. Thus, the first aim of the present study is to test this possibility using the patch-clamp technique in the isolated mouse PT.

It is well established that adenosine is an important factor which regulates tubule-glomerular feedback mechanism (Vallon et al., 2004; Carlstrom et al., 2010), renin secretion and renal membrane transport process via adenosine receptors (Vallon et al., 2006; Welch, 2015). Adenosine has been shown to be generated during renal tubular transport process and ecto-5′-nucleotidase in the brush-border membrane of the PT is responsible for converting ATP, ADP and AMP to the extracellular adenosine which can then activate adenosine receptors in the PT (Siegfried et al., 1996). While ecto-5′-nucleotidase is predominantly expressed in the apical membrane of PT, Unwin’s group has shown that ecto-5′-nucleotidase is expressed also in the peritubular side to a lesser degree (Vekaria et al., 2006). Although there are four types of adenosine receptors in the kidney, A1, A2a, A2b, A3, (Di Sole, 2008), adenosine-A1, adenosine-A2a and adenosine-A2b receptors have been confirmed to be expressed in the PT (Wengert et al., 2005; Vallon et al., 2006). The role of adenosine-A1 receptor in regulating trans-epithelial Na^+^ transport of the PT was demonstrated by experiments in which the stimulation of adenosine-A1 receptor increased Na^+^ transport via NHE_3_ in the PT (Takeda et al., 1993). However, the effect of adenosine on Na^+^ transport in the PT via adenosine-A1 receptor has been shown to be a biphasic effect, a low concentration stimulates whereas a high concentration inhibits trans-epithelial Na^+^ transport of PT (Babich et al., 2015). The role of adenosine-A1 receptor in the stimulation of PT Na^+^ transport was also suggested by the finding that the inhibition of adenosine-A1 receptor increased renal natriuresis without K^+^ wasting (Vallon et al., 2006). As mentioned above, the basolateral K^+^ channel of the PT is involved in regulating trans-epithelial Na^+^ transport (Bignon et al., 2020). Thus, it raises the possibility that adenosine may also modulate the basolateral K^+^ channel activity thereby affecting NHE_3_ function. Accordingly, the second aim of the present study is to explore whether adenosine regulates the basolateral K^+^ channel of the PT and to determine the adenosine receptor and the signaling pathway which mediates the effect of adenosine on the K^+^ channels.

## Materials and methods

### Preparation of renal proximal tubule

Pathogen-free C57BL/6J mice of either sex, 3–5 weeks old, were provided by the Experimental Animal Center of Xuzhou Medical University. After cervical dislocation, the abdomen of the mouse was opened, and the kidneys were taken out. The renal capsule was removed first, and the cortical sections with a thickness of about 0.5 mm was cut with a thin blade. The renal tissue was digested in a collagenase (1 mg/1 mL) containing bath solution at 37°C for 40–50 min. Proximal tubules were dissected under a dissecting microscope and the isolated tubules were transferred onto a 6 × 6-mm cover glass coated with polylysine. We mainly used the late convoluted portion of PT before straight portion of the PT (late S2) and S3 segment for the experiments. However, we have also used early portion of the PT (100 μm after glomerulus, S1) for the patch-clamp experiments. After dissection, the PT on the cover glass was placed in a chamber mounted on an inverted microscope.

## Ethical approval

The protocol for animal use has been approved by independent Institutional Animal Use and Care Committee at Xuzhou Medical University.

### Single-channel patch-clamp recordings

The glass microelectrodes for the patch-clamp experiment were pulled in two steps by PC-100 microelectrode-puller (sharinge, Japan). After the electrode was filled with the pipette solution, the pipette resistance in the bath solution was 10–20 MΩ. We selected smoothly-looking PT, a sign of healthy tubule, for the single channel recording. An Axopatch 200B amplifier and a Digidata 1,322 interface (Molecular Devices, United States) were used for single-channel recordings. Single-channel currents were recorded in cell-attached mode using Clampex 10.7 recording software (Molecular Devices, United States). The basolateral membrane of the PT was sealed to form a high resistance seal (>1 GΩ), and then single-channel recordings were implemented.

Data were analyzed by Clampfit software system 10.7 (Molecular Devices,United States). NP_o_ is regarded as an index for the activity of K^+^ channels. NP_o_ is the product of the channel open probability (P_o_) and channel number (N). The NP_o_ was calculated by the formula

$${NP}_{o}=\Sigma \;\left(1t_{1}+2t_{2}+\ldots +{it}_{i}\right),$$

where i refers to the number of current level, and ti is the fractional open time spent at each of the observed current levels. For calculating the channel slope conductance, the channel currents were measured at several holding potentials. For studying the effect of adenosine on the K^+^ channel, the holding potential was set at 0 mV in cell-attached mode.

### Experimental solution and chemicals

Bath solution (mM): NaCl 140, KCl 5, MgCl_2_ 1.8, CaCl_2_ 1.8, HEPES 10 (pH = 7.4). Pipette solution (mM): KCl 140, MgCl2 1.8, HEPES 10 (pH7.4). Collagenase II, DPCPX, CP-66713, AACOCF3, Calphostin C, U73122, H-8 (N—[2- (methylamino) ethyl] -5- isoquinoline sulfonamide-2HC1), and the chemical reagent used for preparation of above solution were all purchased from Sigma. AACOCF3, Calphostin C and U73122 are first dissolved in dimethyl sulfoxide (DMSO), and then diluted to the required concentration with bath solution. The final concentration of DMSO in the bath does not exceed 0.1%, which has no effect on channel activity. H-8 is directly prepared with a bath solution.

### Experimental statistics

The data were presented as means ± SEM. We used SPSS20.0 software to conduct statistical analysis of the data. Student’s paired *t*-test was used to determine the significance of value difference before and after drug treatment, and *p* < 0.05 indicated that the difference was statistically significant.

## Results

### Inwardly-rectifying 50 pS K^+^ channel is the predominant type K^+^ channel in the basolateral membrane of the PT

**FIGURE 1:**
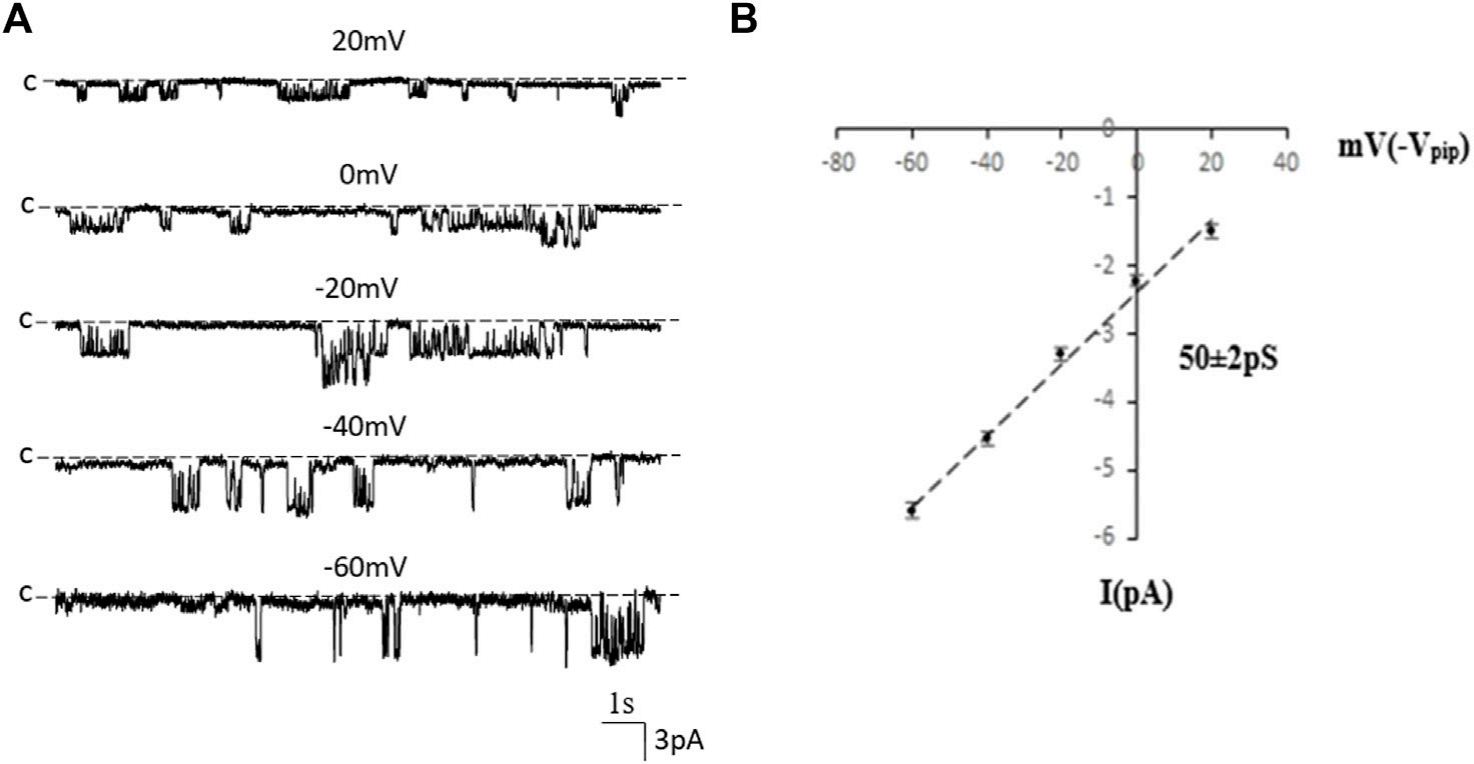
The 50 pS inwardly-rectifying K^+^ channel is a predominant type of the basolateral K^+^ channel of the PT. **(A)** A single channel recording made in a cell-attached patch shows the channel activity at different membrane potentials. “C” indicates that the channel close level. **(B)** A current (I)/voltage (V) relationship curve shows that the slope conductance of the K^+^ channel is 50 pS.

**FIGURE 2:**
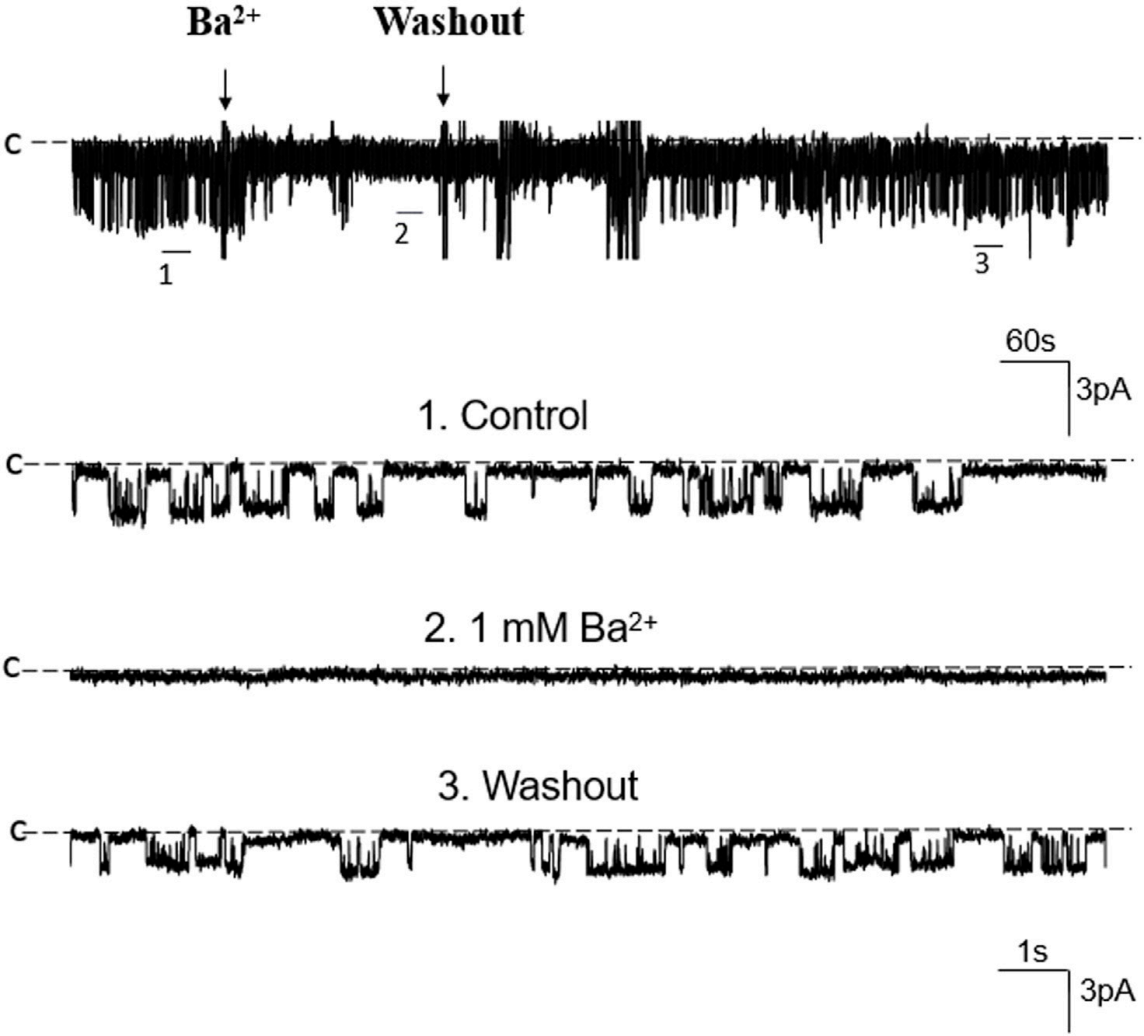
Ba^2+^ inhibits the 50 pS K^+^ channel of the PT. A single channel recording made in an inside-out patch shows that Ba^2+^ (1 mM) inhibits the 50 pS K^+^ channel activity. Three parts of the trace indicated by numbers are extended to show the fast time resolution. Holding potential is at 0 mV “C” indicates that the channel close level.

We first used single-channel-recording technique to examine K^+^ channel in the basolateral membrane of PT. From total 1,336 patches with high resistance (>GΩ), we have detected K^+^ channel activity in 651 patches (48.7%). Furthermore, from these 651 patches performed on S1, S2 and S3 segments, we have detected a 50 pS K^+^ channel in total 586 patches (90%) (The average channel number per patch was about 2). Figure 1A is a typical channel recording showing the 50 pS K^+^ channel (in cell-attached model) at different voltages and Figure 1B is the current/voltage curve yielding a conductance of 50 ± 2 pS between −60 and −20 mV (*n* = 5). This 50 pS K^+^ channel was inhibited by Ba^2+^. Figure 2 is a channel recording made in inside-out patches showing that adding 1 mM Ba^2+^ inhibits the 50- pS K^+^ channel. Thus, our data indicate that the 50 pS K^+^ channel is predominant K^+^ channel type in the basolateral membrane of the mouse PT.

### Adenosine stimulates basolateral 50 pS K^+^ channels in PT

**FIGURE 3:**
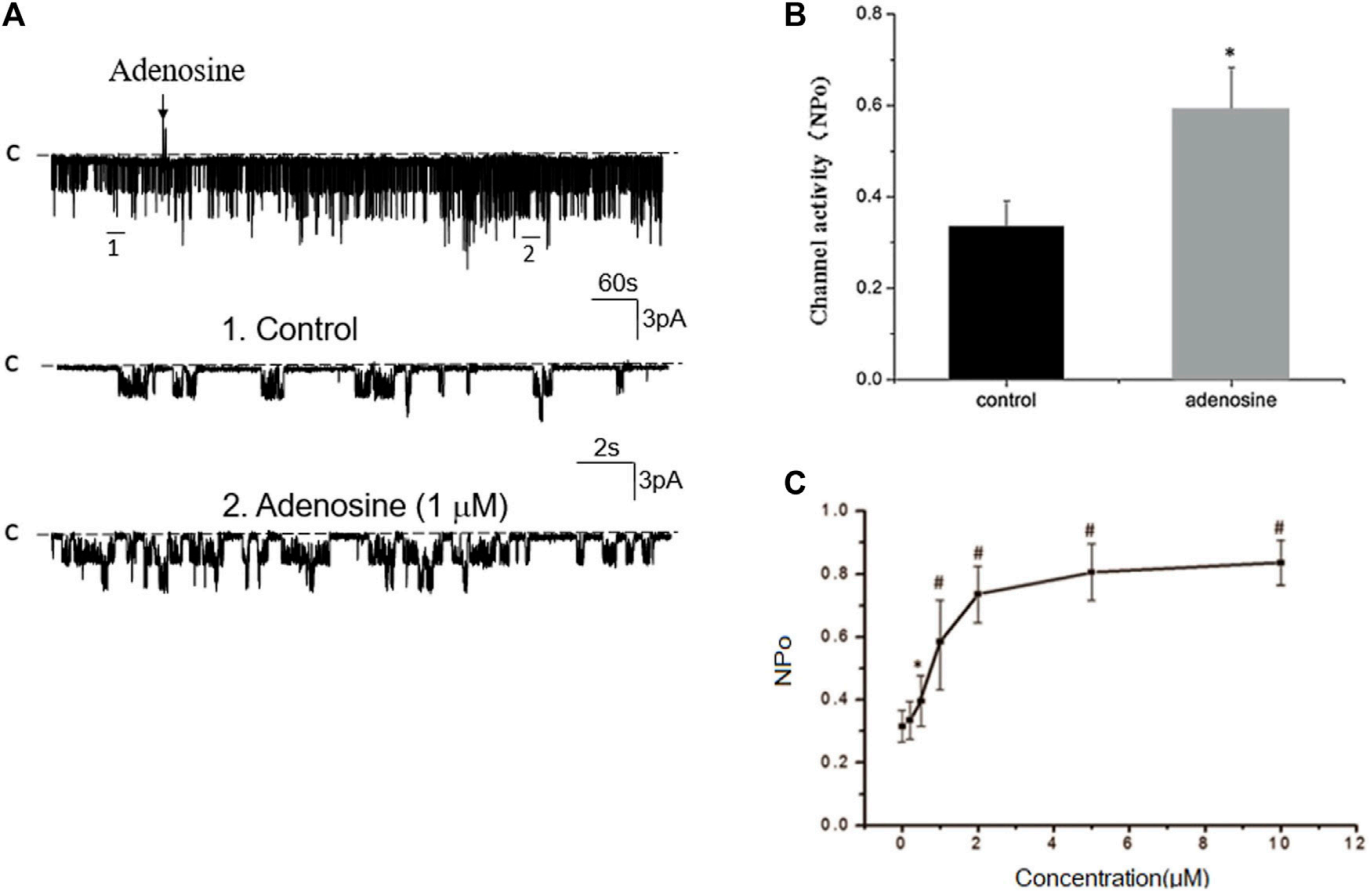
Adenosine stimulates the basolateral 50 pS K^+^ channel of PT. **(A)** The top trace shows the time course of the affect of adenosine (1 μM) on the 50 pS K^+^ channel in a cell-attached patch. Two parts of the trace indicated by numbers are extended to show the fast time resolution. Holding potential is at 0 mV “C” indicates that the channel close level. **(B)** A bar graph summarizes the effect of 1 μm adenosine and adenosine-A1 receptor agonist on the 50 pS K^+^ channel. **(C)** A dose-response curve shows the effect of adenosine on the 50 pS K^+^ channel of the PT. *p < 0.05, #*p* < 0.01, compared with control (0 μM).

We next examined whether adenosine was able to stimulate this 50 pS K^+^ channel in the PT. Figure 3A is a typical recording showing the effect of adenosine on the basolateral 50 pS K^+^ channel of the PT in a cell-attached patch. After detecting the K^+^ channel activity, we recorded the K^+^ channel activity for 5 min as the control value and then adding 1 μM of adenosine to the bath while the 50 pS K^+^ channel activity was continuously monitored for an additional 10–15 min. The results are summarized in a bar graph showing that adenosine stimulates the 50 pS K^+^ channel (Figure 3B). Figure 3C is a dose response curve of the effect of adenosine on the 50 pS K^+^ channel. We observed that adenosine at 500 nM was able to significantly increase the 50 pS K^+^ channel activity, defined by NP_o_ from 0.33 ± 0.05 to 0.43 ± 0.05 whereas adding 1 μM adenosine increased NP_o_ from 0.33 ± 0.05 to 0.59 ± 0.09 (*n* = 9, *p* < 0.01). Adding 2 μM adenosine stimulated K^+^ channel and increased NP_o_ to 0.7 ± 0.09, a value close to maximal stimulation. Since 1 μM adenosine-induced stimulation of the 50 pS K^+^ channel has reached to a submaximal level, we have used 1 μM adenosine in the following experiments. Although the concentration of adenosine (1 μM) was higher than the physiological ranges, the aim of the experiments is to prove the principle that adenosine stimulates the basolateral 50 pS K^+^ channels in the PT.

### Adenosine A1 receptor mediates the effect of adenosine on the 50 pS K^+^ channel

**FIGURE 4:**
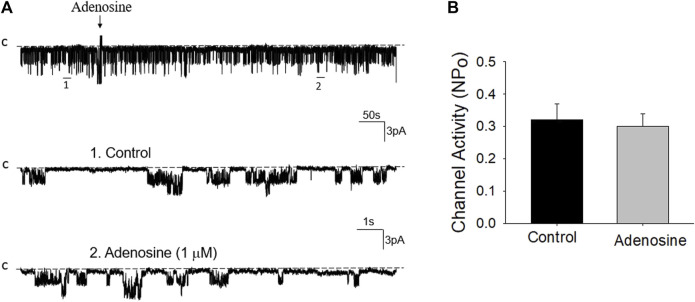
Inhibition of adenosine-A1 receptor abolished the effect of adenosine on the basolateral 50 pS K^+^ channel of the PT. **(A)** A single channel recording (in a cell-attached patch) shows the time course of adenosine effect (1 μm) on the 50 pS K^+^ channel in the presence of DPCPX, an adenosine-A1 receptor antagonist. The experiments were performed in the PT pretreated with 10 μm DPCPX (which was continuously present in the bath). Two parts of the trace indicated by numbers are extended to show the fast time resolution. Holding potential is at 0 mV “C” indicates that the channel close level. **(B)** A Bar graph summarizes the effect of adenosine on the 50 pS K^+^ channel in the presence of DPCPX.

**FIGURE 5:**
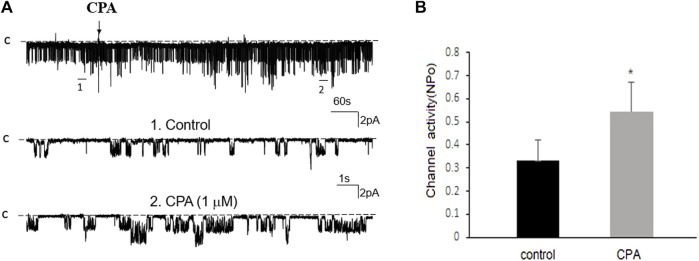
Adenosine-A1 receptor agonist stimulates the basolateral 50 pS K^+^ channel of PT. **(A)** The top trace shows the time course of the affect of 1 μm N6-cyclopentyladenosine (CPA) on the 50 pS K^+^ channel in a cell-attached patch. Two parts of the trace indicated by numbers are extended to show the fast time resolution. Holding potential is at 0 mV “C” indicates that the channel close level. **(B)** A bar graph summarizes the effect of 10 μm CPA on the 50 pS K^+^ channel.

**FIGURE 6:**
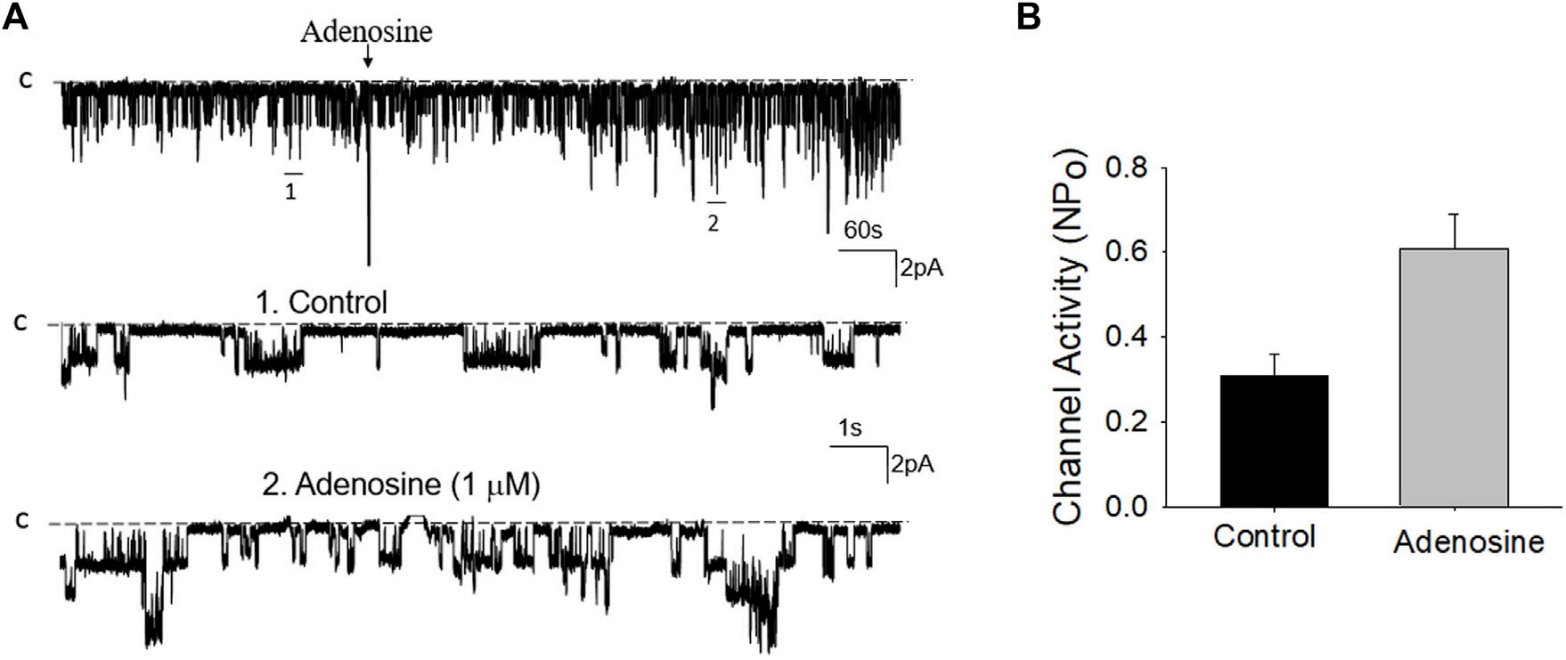
Inhibition of adenosine-A2 receptor fails to abolish the effect of adenosine on the basolateral 50 pS K^+^ channel of the PT. **(A)** A single channel recording (in a cell-attached patch) shows the time course of adenosine effect (1 μm) on the 50 pS K^+^ channel in the presence of 10 μm CP-66713, an adenosine A_2_ receptor antagonist. The experiments were performed in the PT pretreated with CP-66713 (which was continuously present in the bath). Two parts of the trace indicated by numbers are extended to show the fast time resolution. Holding potential is at 0 mV “C” indicates that the channel close level. **(B)** A Bar graph summarizes the effect of adenosine on the 50 pS K^+^ channel in the presence of CP-66713.

Previous studies have shown that adenosine-A1, -A2a and -A2b receptors are expressed in the PT (Wengert et al., 2005; Vallon et al., 2006). Moreover, both adenosine-A1 and -A2a receptors have been shown to regulate Na^+^ transport in the PT (Di Sole et al., 2002; Wengert et al., 2005; Di Sole, 2008; Babich et al., 2015). Thus, we next examined whether adenosine-A1 or -A2a receptor mediates the effect of adenosine on the 50 pS K^+^ channel in PT. We examined the effect of adenosine on the 50 pS K^+^ channel in the PT pretreated with DPCPX (inhibitor of adenosine A1 receptor) or CP-66713 (inhibitor of adenosine-A2 receptor) for 10–15 min. The reason that we could not perform experiments in which the 50 pS K^+^ channel activity was continuously monitored after adding either DPCPX or CP-66713 and then adenosine is due to technical difficulty to patch the PT cells for more than 20 min. Figure 4A is a typical single channel recording showing the effect of adenosine on the 50 pS K^+^ channel in the PT pretreated with 10 μM DPCPX (which was still present in the bath). The results are summarized in a bar graph (Figure 4B). It is apparent that adenosine failed to stimulate the basolateral 50 pS K^+^ channels in the presence of DPCPX (control, 0.32 ± 0.05; adenosine 0.29 ± 0.04, *n* = 6), suggesting that the stimulatory effect of adenosine on 50 pS K^+^ channels was mediated by adenosine-A1 receptors. This notion is also supported by the finding that adenosine-A1 agonist, CPA, is able to mimic the effect of adenosine on the 50 pS K^+^ channel activity. Figure 5A is a single channel recording showing the effect of CPA (1 μM) on the 50 pS K^+^ channel in the PT and Figure 5B is a bar graph summarizing the results. CPA increased the NP_o_ of the 50 pS K^+^ channel from 0.34 ± 0.05 to 0.55 ± 0.09 (*n* = 6, *p* < 0.01). Although data support the notion that stimulation of adenosine-A1 receptor activate the 50 pS K^+^ channel of the PT, further experiments are required to explore whether CPA has a dual effect on the 50 pS K^+^ channel. The notion that the effect of adenosine on the K^+^ channel was mediated by adenosine-A1 receptor was also suggested by experiments in which the effect of adenosine on the 50 pS K^+^ channel was examined in the presence of adenosine-A2 inhibitor. Figure 6A is a typical single channel recording showing the effect of adenosine on the 50 pS K^+^ channel in the PT pretreated with 10 μM CP-66713 and Figure 6B is a bar graph summarizing the results. It is apparent that adenosine was still able to stimulate the basolateral 50 pS K^+^ in the PT pretreated with 10 μM CP-66713 (which was still present in the bath) (control, 0.31 ± 0.05; adenosine 0.61 ± 0.08, *n* = 6). This suggests that the activation of adenosine on the 50 pS K^+^ channel in the basolateral membrane of the PT was not mediated by adenosine-A2 receptors.

### PLC-PKC pathway, but not PLA2 or PKA, mediates the effect of adenosine on the 50 pS K^+^ channel

**FIGURE 7:**
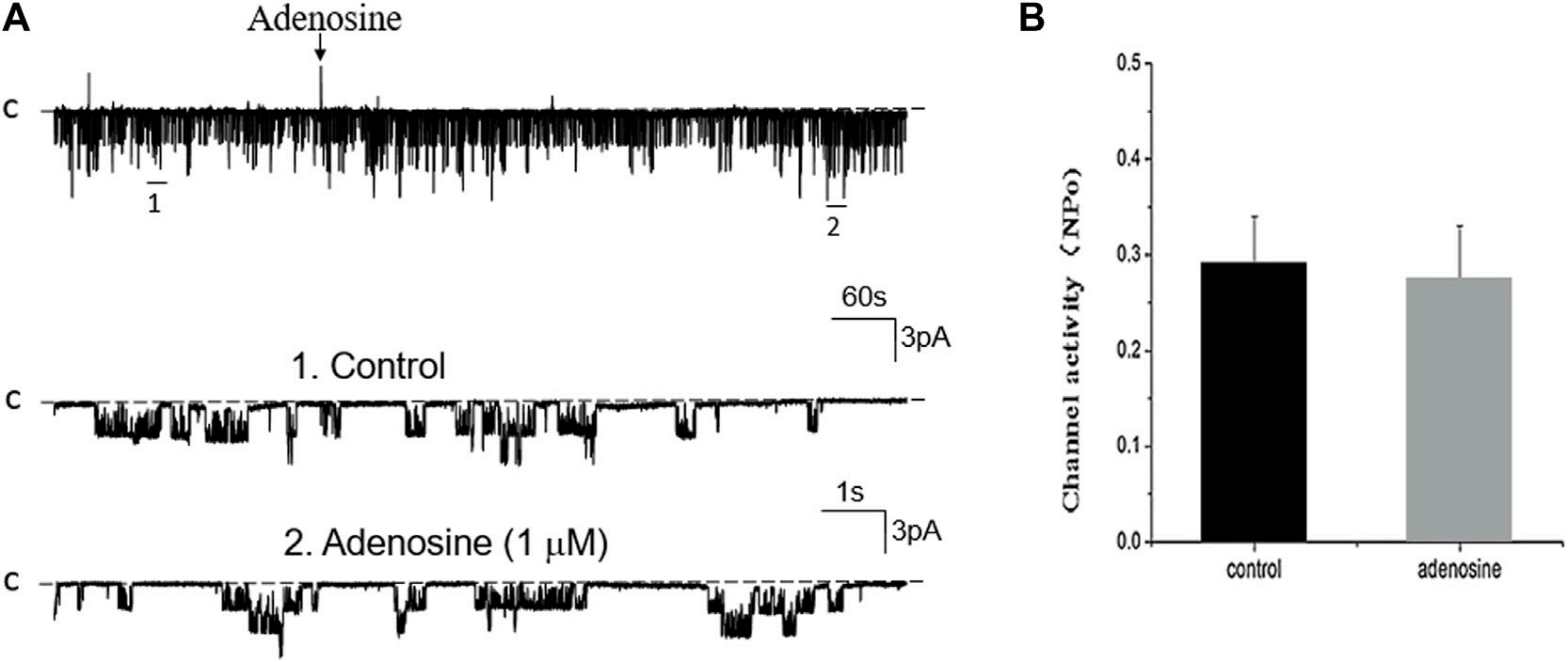
Inhibition of PLC abolishes the effect of adenosine on the basolateral 50 pS K^+^ channel of the PT. **(A)** A single channel recording (in a cell-attached patch) shows the time course of adenosine effect (1 μm) on the 50 pS K^+^ channel in the presence of 10 μm U-73222, a PLC inhibitor. The experiments were performed in the PT pretreated with U-73222 (which was continuously present in the bath). Two parts of the trace indicated by numbers are extended to show the fast time resolution. Holding potential is at 0 mV “C” indicates that the channel close level. **(B)** A Bar graph summarizes the effect of adenosine on the 50 pS K^+^ channel in the presence of U-73222.

**FIGURE 8:**
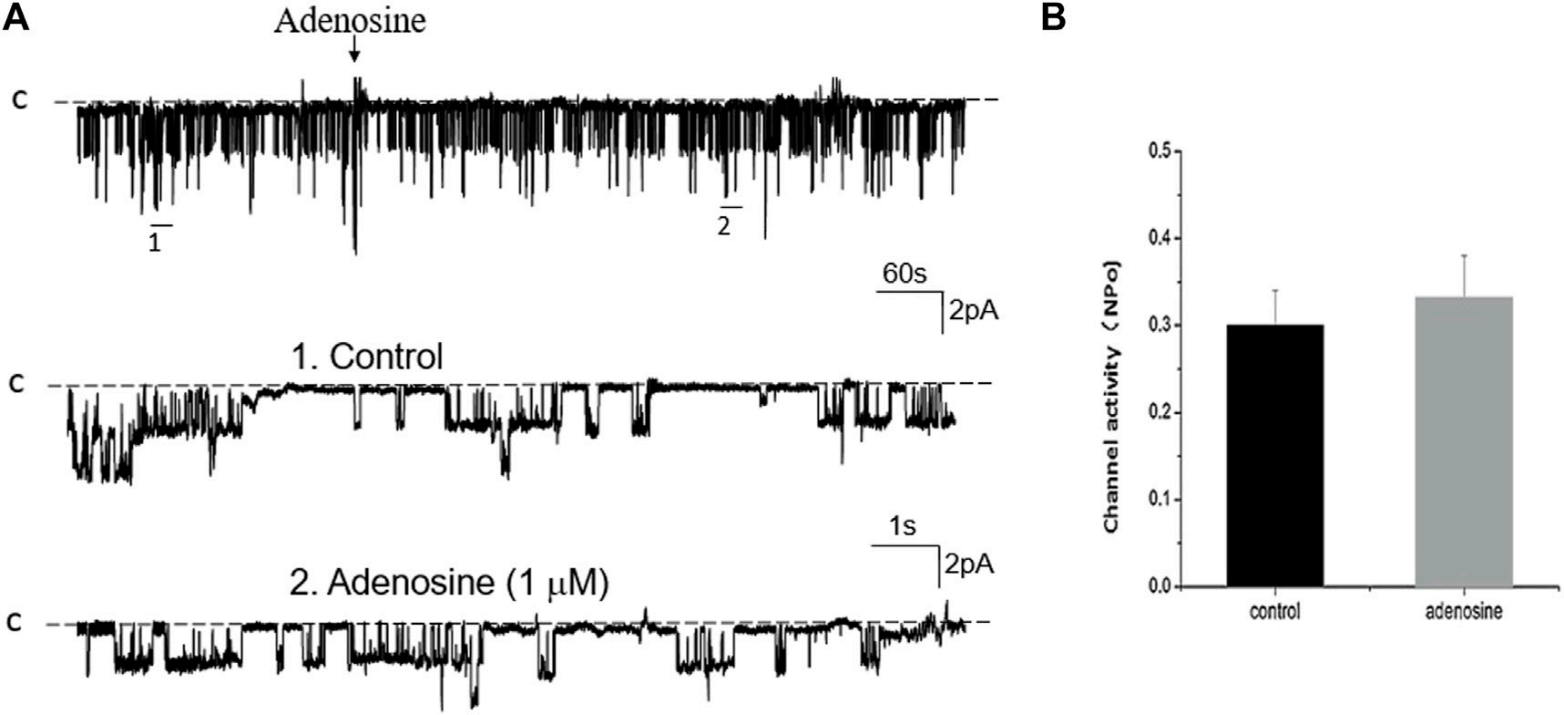
Inhibition of PKC abolishes the effect of adenosine on the basolateral 50 pS K^+^ channel of the PT. **(A)** A single channel recording (in a cell-attached patch) shows the time course of adenosine effect (1 μm) on the 50 pS K^+^ channel in the presence of 100 nM Calphostin C, a PKC inhibitor. The experiments were performed in the PT pretreated with Calphostin C (which was continuously present in the bath). Two parts of the trace indicated by numbers are extended to show the fast time resolution. Holding potential is at 0 mV “C” indicates that the channel close level. **(B)** A Bar graph summarizes the effect of adenosine on the 50 pS K^+^ channel in the presence of Calphostin C.

Adenosine-A1 receptor belong to a G-protein-coupled family. It has been reported that the stimulation of adenosine-A1 receptor may activate three signal transduction pathways (Vallon et al., 2006): Cyclic monophosphate-protein kinase A (cAMP-PKA), phospholipase C-protein kinase C (PLC-PKC) pathway, and phospholipase A2-arachidonic acid (PLA2-AA). We then examined the effect of adenosine on the 50 pS K^+^ channel in the PT pretreated with 10 μM U-73122 (phospholipase C inhibitor) or 100 nM Calphostin C (PKC inhibitor) for 10–15 min. Figure 7A is a single channel recording showing the effect of adenosine on the 50 pS K^+^ channel in the PT pretreated with U-73122 and Figure 7B is a bar graph summarizing the results. Inhibition of PLC was able to abolish the effect of adenosine on the K^+^ channels, suggesting that PLC mediates the effect of adenosine on the 50 pS K^+^ channel in the basolateral membrane of the PT. Similar to U-73122, the inhibition of PKC also abolished the effect of adenosine on the 50 pS K^+^ channel activity. Figure 8A is a typical single channel recording showing the effect of adenosine on the 50 pS K^+^ channel in the PT pretreated with 100 nM of Calphostin C and Figure 8B is a bar graph summarizing the results. It is apparent that the inhibition of PKC also abolished the effect of adenosine on the 50 pS K^+^ channel.

**FIGURE 9:**
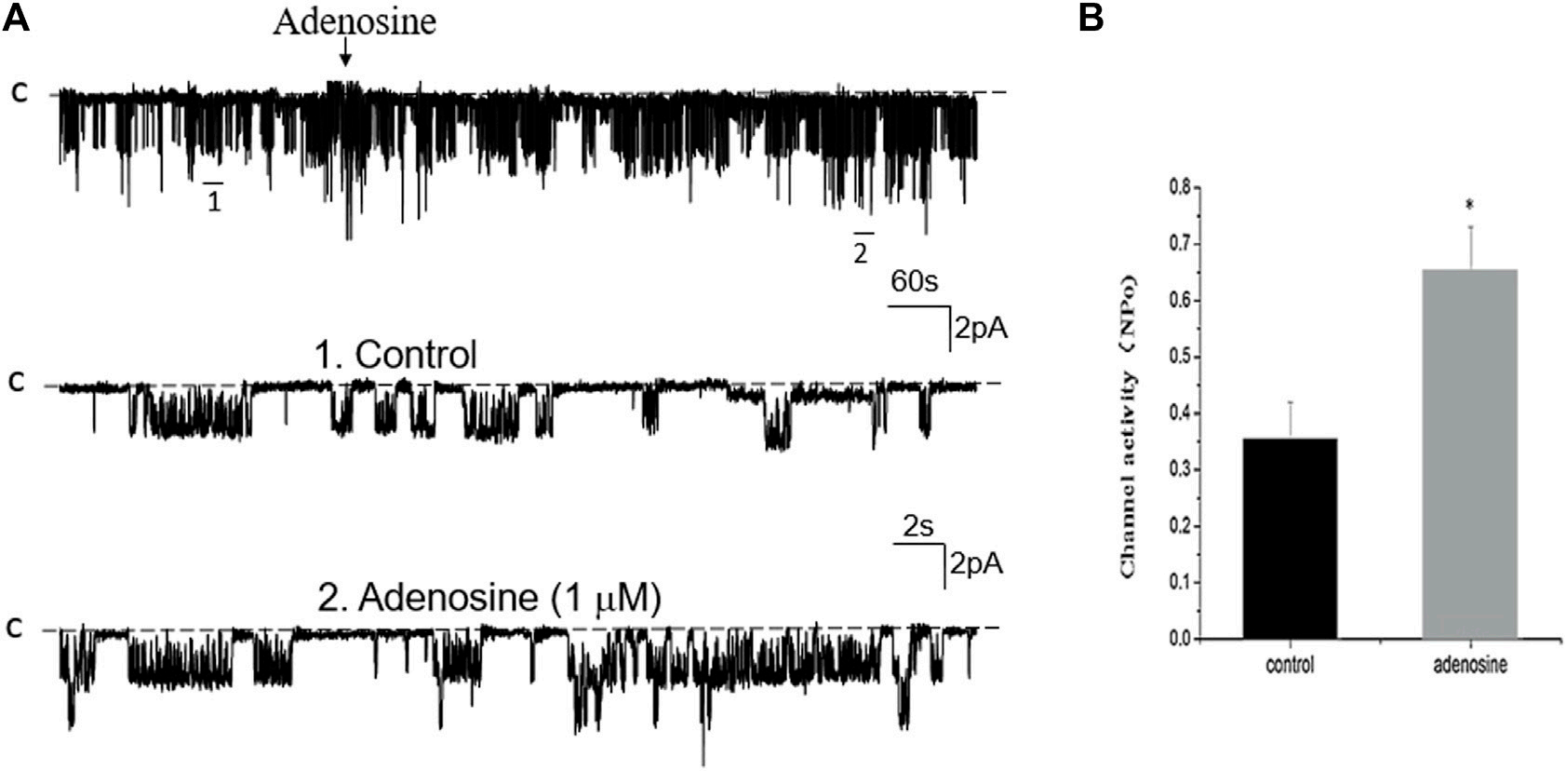
Inhibition of PLA_2_ fails to abolish the effect of adenosine on the basolateral 50 pS K^+^ channel of the PT. **(A)** A single channel recording (in a cell-attached patch) shows the time course of adenosine effect (1 μm) on the 50 pS K^+^ channel in the presence of 10 μm AACOCF3, an inhibitor of PLA_2_. The experiments were performed in the PT pretreated with AACOCF3 (which was continuously present in the bath). Two parts of the trace indicated by numbers are extended to show the fast time resolution. Holding potential is at 0 mV “C” indicates that the channel close level. **(B)** A Bar graph summarizes the effect of adenosine on the 50 pS K^+^ channel in the presence of AACOCF3.

**FIGURE 10:**
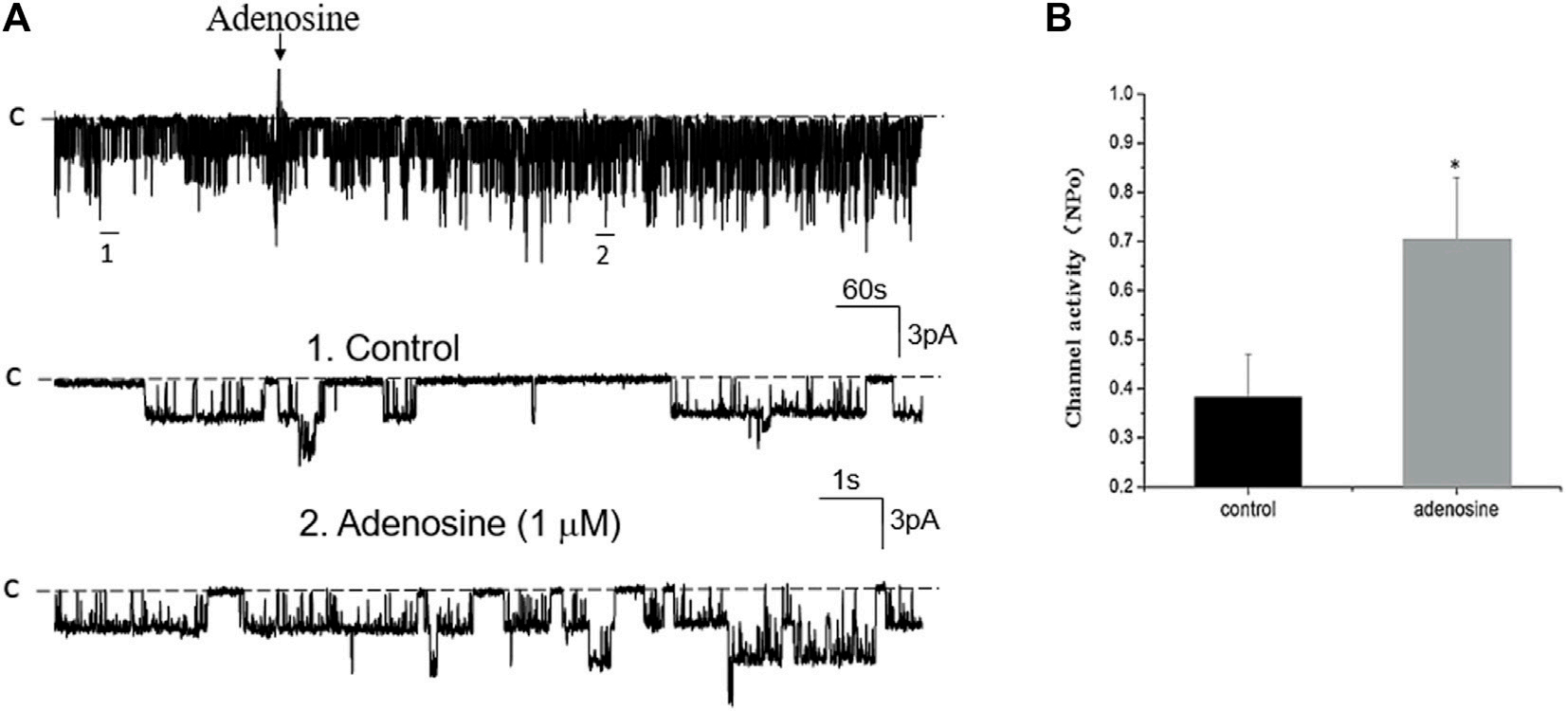
Inhibition of PKA fails to abolish the effect of adenosine on the basolateral 50 pS K^+^ channel of the PT. **(A)** A single channel recording (in a cell-attached patch) shows the time course of adenosine effect (1 μm) on the 50 pS K^+^ channel in the presence of 1 μm H8, an inhibitor of PKA. The experiments were performed in the PT pretreated with H8 (which was continuously present in the bath). Two parts of the trace indicated by numbers are extended to show the fast time resolution. Holding potential is at 0 mV “C” indicates that the channel close level. **(B)** A Bar graph summarizes the effect of adenosine on the 50 pS K^+^ channel in the presence of H8.

We next examined the effect of adenosine on the 50 pS K^+^ channel in the PT pretreated with 10 μM AACOCF3 (phospholipase A2 inhibitor) or 1 μM H8 of (PKA inhibitor). Figure 9A is a typical single channel recording showing the effect of adenosine on the 50 pS K^+^ channel in the PT pretreated with 10 μM AACOCF3 and Figure 9B is a bar graph summarizing the results. It is apparent that 1 μM adenosine still activated the 50 pS K^+^ channel of the PT. Figure 10A is a single channel recording showing the effect of adenosine on the 50 pS K^+^ channel in the PT pretreated with 1 μM H8 and Figure 10B is a bar graph summarizing the results. In the presence of H8, adenosine still stimulated the basolateral 50 pS K^+^ channel in the PT. Taken together, data strongly suggest that the stimulatory effect of adenosine on the 50 pS K^+^ channel was mediated by PLC-PKC pathway but not by PLA2 or PKA pathway.

## Discussion

We have used the single channel recording to detect a 50 pS inwardly-rectifying K^+^ channel in the basolateral membrane of the PT. The renal PT is responsible for the complete reabsorption of filtered glucose and amino acids and is also responsible for reabsorption of 65%–70% filtered Na^+^, Cl^−^, K^+^, and water, as well as about 80% of HCO_3_^−^ (Aronson and Sacktor, 1975; Aronson, 1983; 1989) Classic role of the basolateral K^+^ channels of the PT is considered to be responsible for the K^+^ recycling across the basolateral membrane of the PT to sustain the Na^+^-K^+^-ATPase activity, so-called pump-leaky mechanism (Schultz, 1981). However, the recent development in the field has also suggested the possibility that the basolateral K^+^ channels of the PT are also involved in the regulation of ion transporters other than Na^+^-K^+^-ATPase (Bignon et al., 2020; Lin et al., 2022). Two lines of evidence have strongly suggested that this 50 pS K^+^ channel is most likely composed of Kir4.2 (encoding by *Kcnj15)* and Kir5.1 (encoding by *Kcnj16*) which are known to expressed in the basolateral membrane of the mouse PT and are responsible for the basolateral K^+^ conductance (Tucker et al., 2000; Bignon et al., 2020; Lin et al., 2022). First, this 50 pS K^+^ channel is absent in Kir5.1 knockout mice (Wen-Hui Wang’s personal communication); Second, the 50 pS K^+^ channel is a predominant type of basolateral K^+^ channel in the mouse PT. This is in consistence of the finding that Kir4.2/Kir5.1 heterotetramer is a predominant type K^+^ channel in the basolateral membrane of the mouse PT (Bignon et al., 2020), because deleting either Kir4.2 or Kir5.1 depolarizes PT membrane. Our finding is also consistent with the previous observation that a 50-pS to 60-pS inwardly-rectifying K^+^ channel is a main type of basolateral K^+^ channel in the rabbit PT (Tsuchiya et al., 1992; Beck et al., 1993; Noulin et al., 1999).

The main finding of the present study is to demonstrate that adenosine stimulates the basolateral 50 pS K^+^ channel in the PT via adenosine-A1 receptor and that the effect of adenosine on the K^+^ channel is mediated by PLC-PKC signaling pathway. This notion is supported by three lines of evidences: 1) Adenosine-induced stimulation of the 50 pS K^+^ channel was absent in the presence of adenosine-A1 receptor inhibitor; 2) Adenosine-A1 receptor agonist was able to mimic the effect of adenosine on the 50 pS K^+^ channel; 3) Inhibition of either PLC or PKC but not PKA or PLA2 abolished the effect of adenosine on the 50 pS K^+^ channel in the PT. Although we have used a higher adenosine concentration than a physiologically relevant concentration in our study, we have also observed that adenosine at 500 nM was able to stimulate the 50-pS K^+^ channel. Moreover, further experiments are required to explore the mechanism by which PKC regulates the 50 pS K^+^ channel in the PT. The basolateral K^+^ channel of the PT plays an important role in maintaining the trans-epithelial Na^+^ transport in the PT. This notion was convincing demonstrated by the previous report that deletion of Kir4.2, a major Kir channel in the basolateral membrane of the PT (Bignon et al., 2020), impairs trans-epithelial HCO_3_^−^ absorption, presumably by inhibiting NHE_3_. The mechanism by which manipulation of the basolateral K^+^ channel activity affects trans-epithelial HCO_3_^−^ transport of the PT is due to modulation of the basolateral membrane potential. The trans-epithelial Na^+^-HCO_3_^-^ transport is a two-step process: 1) Na^+^ enters the PT cell through apical NHE_3_ and HCO_3_^−^ enters the PT cell through CO_2_ diffusion (Baum, 1987; Boyarsky et al., 1988); 2) Na^+^ is then pumped out of cell through Na^+^-K^+^-ATPase while HCO_3_^−^ is then left the PT cell via an electrogenic Na^+^-HCO_3_^-^ cotransporter (NBCe1) in the basolateral membrane (Romero et al., 1998; Romero and Boron, 1999). Although HCO_3_^−^ exit across the basolateral membrane is also by Cl^−^/HCO_3_^−^ exchanger (Soleimani, 2013), NBCe1 plays a role in HCO_3_^−^ exit. Because the stoichiometry of NBCe1 in the PT is most likely to be 1Na^+^ + 1HCO^−^_3_ + 1CO_3_^2−^ (Wu et al., 2022), NBCe1 operation is electrogenic. Thus, it is conceivable that the basolateral membrane potentials is expected to affect NBCe1 function as reported in Kir4.1 knockout mice (Bignon et al., 2020), such that hyperpolarization facilitates whereas depolarization inhibits the function of NBCe1. Thus, the alteration of the basolateral K^+^ channel activity is expected to affect the function of NBCe1. Consequently, hyperpolarization-induced stimulation of NBCe1 should decrease the intracellular HCO_3_^−^ levels thereby acidifying the intracellular pH which should stimulate NHE_3_ function (Aronson et al., 1982). Also, the stimulation of NBCe1 should decrease the intracellular Na^+^ concentration thereby increasing the driving force for NHE_3_ function. In contrast, depolarization-induced inhibition of NBCe1 should increase both intracellular Na^+^ and HCO_3_^−^ concentration. This should inhibit NHE_3_ function due to a reduction of chemical gradient of Na^+^ and alkalization of PT cells. This notion is supported by the finding that deletion of Kir4.2 depolarizes the PT membrane potential and decreased acid-load-induced pH-recovery rate (Bignon et al., 2020), an indication of NHE_3_ inhibition. Since the stimulation of adenosine-A1 receptor increased the 50 pS K^+^ channel activity of the PT, it is conceivable that activation of the basolateral K^+^ channel activity should also stimulate the trans-epithelial Na^+^ and HCO_3_^−^ absorption induced by the stimulation of adenosine-A1 receptor.

**FIGURE 11:**
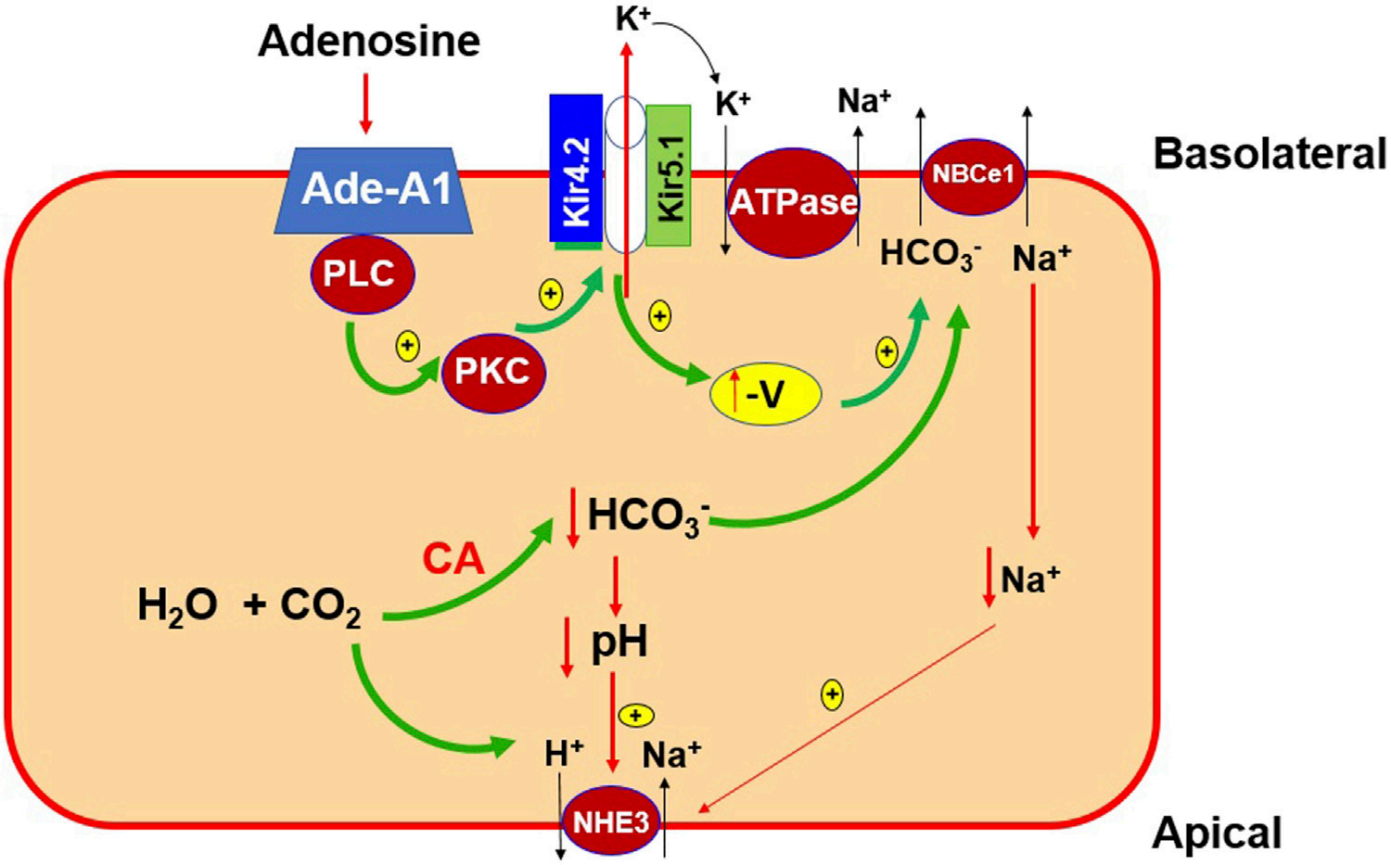
A cell scheme illustrating the role of 50 pS K^+^ channel (a Kir4.2/Kir5.1 heterotetramer) in the regulation of the membrane transport process of the PT. The cell model is a simplified PT cell model rather than a specific PT segment. Abreviations: adenosine-A1 receptor (Ade-A1), phospholipase C (PLC), protein kinase C (PKC), the negativity of PT cell voltage (-V), carbo-anhydrase (CA), electrogenic sodium-bicarbonate cotransport 1 (NBCe1), Na-H-exchanger 3 (NHE_3_).

Figure 11 is a cell scheme illustrating the physiological significance of our finding. This cell model is just a simplified PT cell which does not represent any specific PT segment. Accordingly, many transport proteins such as NHE1 and H^+^-ATPase are not included. This cell model is only used to illustrate the possible role of the basolateral 50 pS K^+^ channel in mediating the effect of adenosine on transepithelial Na^+^ transport in the PT. We hypothesize that the stimulation of adenosine-A1 receptor is expected to modulate trans-epithelial membrane transport of the PT by targeting the basolateral 50 pS K^+^ channel (most likely Kir4.2/Kir5.1 heterotetramer). Activation of the basolateral K+ channel activity by adenosine should facilitate not only Na^+^-K^+^-ATPase activity but also should enhance electrogenic transporter activity such as NBCe1 thereby affecting Na^+^ and HCO_3_^−^ absorption in the PT. It is highly possible that the basolateral Kir4.2/Kir5.1 mat also have an effect on the membrane transport protein other than NBCe1. Further experiments are required to explore the specific role of the 50 pS K^+^ channel in modulating function of the transport protein such as NHE1, H^+^-ATPase or phosphate transport. We conclude that adenosine stimulates the 50 pS K^+^ channels in the basolateral membrane of renal PT via adenosine-A1 receptor and that PLC-PKC signaling pathway mediates the effect of adenosine on the 50 pS K^+^ channel.

## Data Availability

The raw data supporting the conclusion of this article will be made available by the authors, without undue reservation.
