# Supplementary material for: Adenosine stimulates the basolateral 50 pS K+ channel in renal proximal tubule via adenosine-A1 receptor
Source: Front Physiol. 2023 Aug 28;14:1242975. doi: 10.3389/fphys.2023.1242975 (PMC10493268; doi:10.3389/fphys.2023.1242975)
Supplement: Supplementary file 1 [file Presentation1.PPTX]

## Slide 1
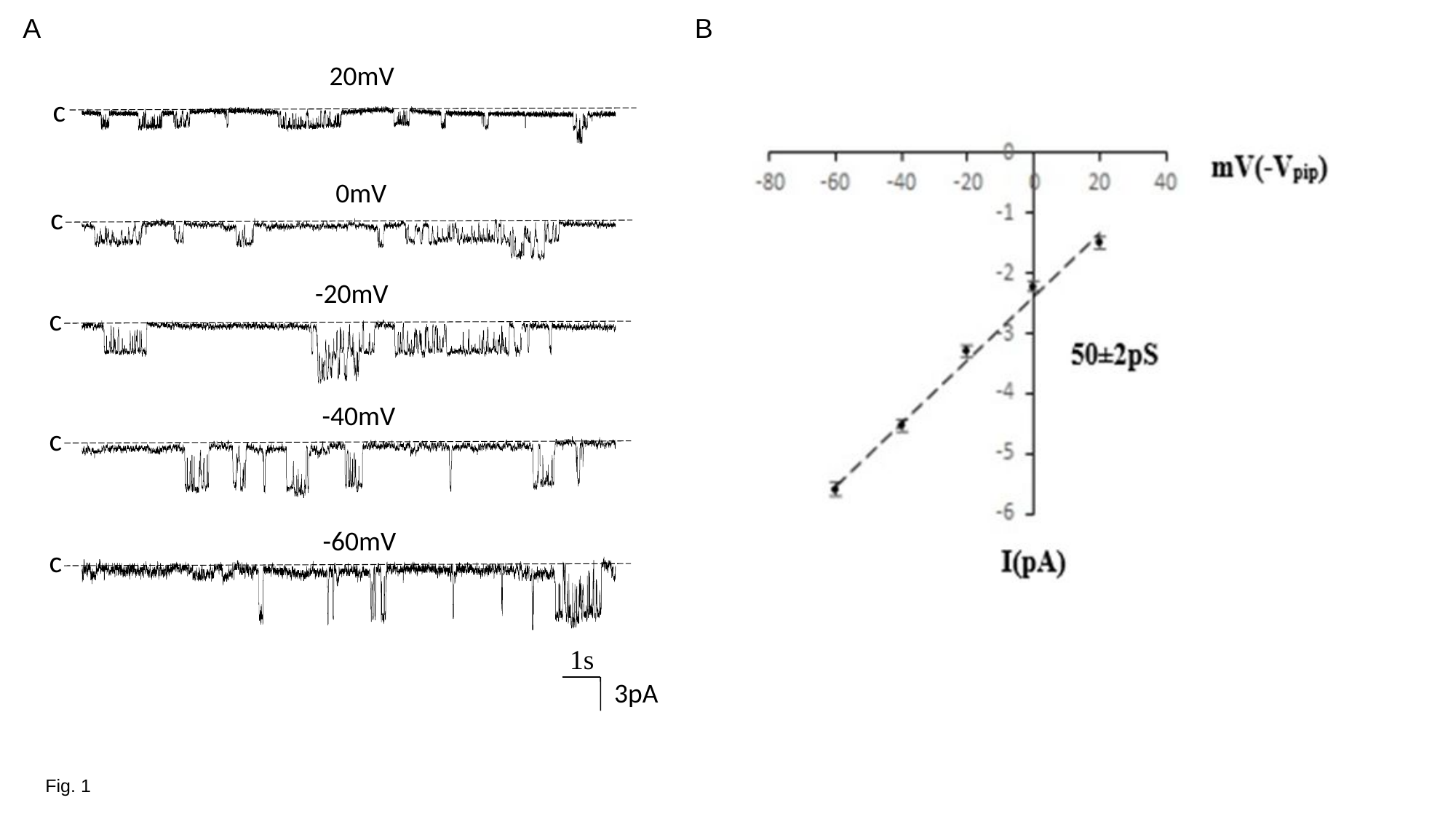

A
 B
20mV
0mV
-20mV
-40mV
-60mV
1s
3pA
c
c
c
c
c
Fig. 1

## Slide 2
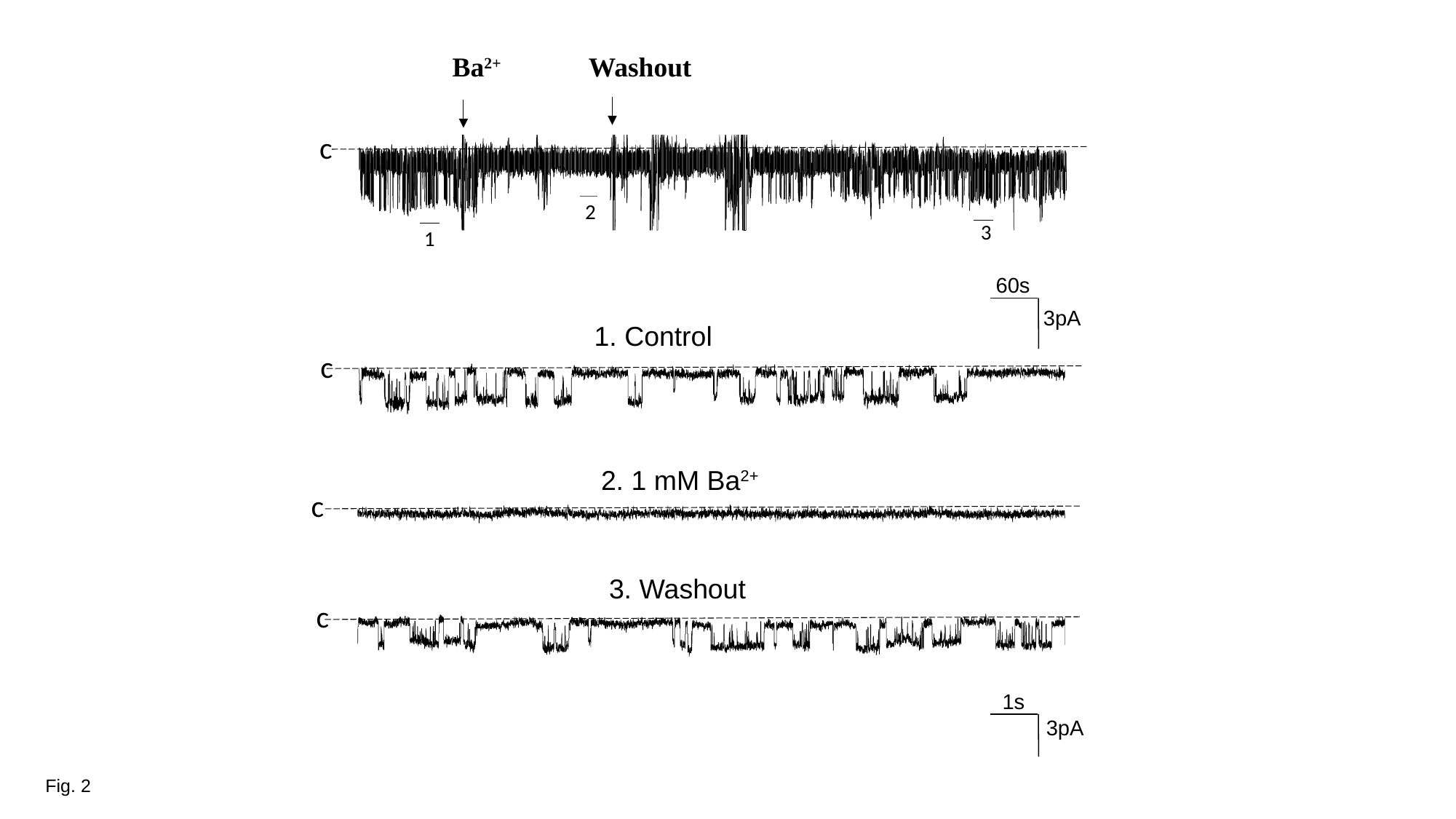

Ba2+
Washout
c
2
3
1
60s
3pA
c
1s
3pA
1. Control
2. 1 mM Ba2+
c
3. Washout
c
Fig. 2

## Slide 3
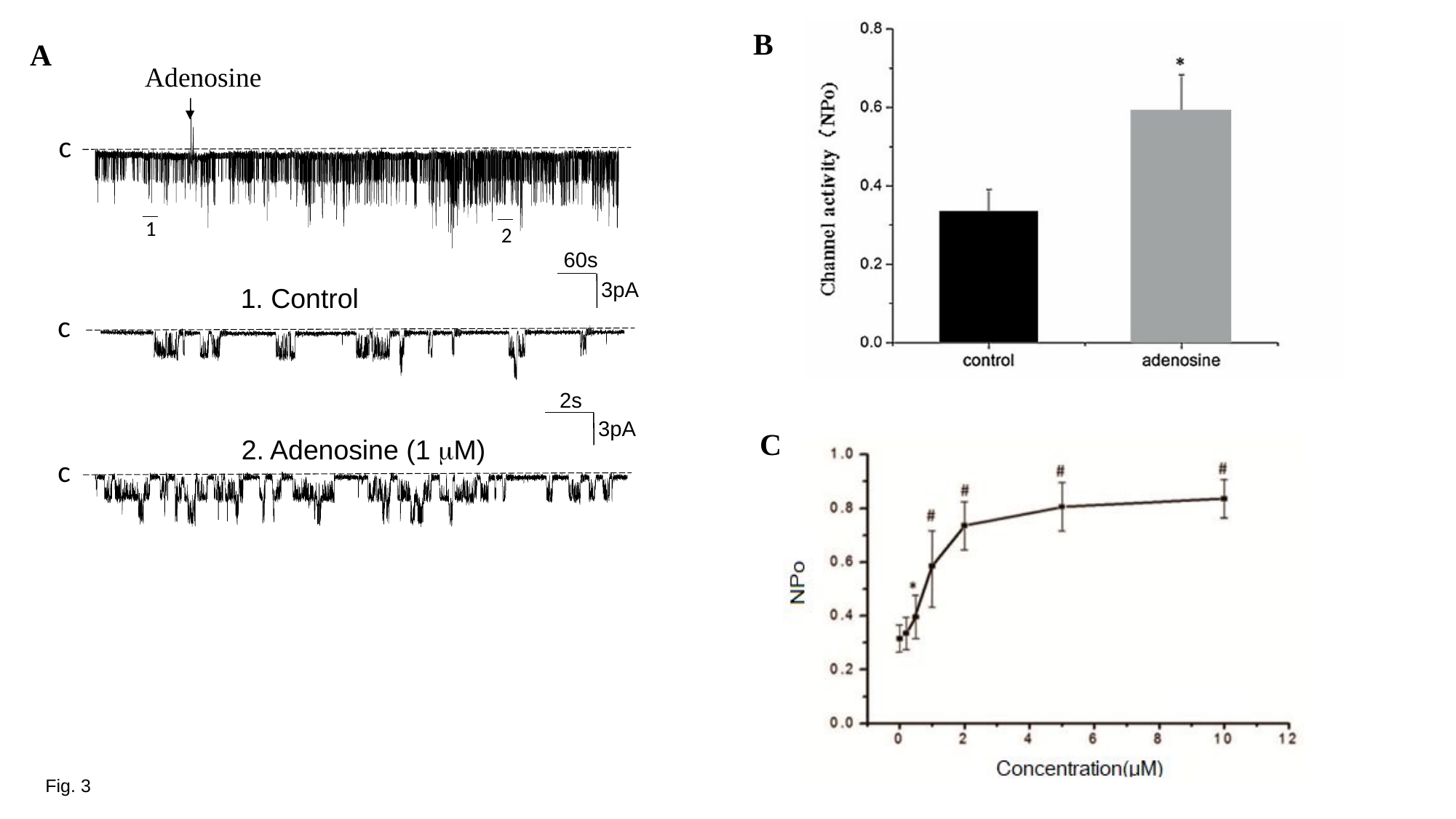

B
 A
Adenosine
c
1
2
60s
3pA
c
2s
3pA
c
1. Control
 C
2. Adenosine (1 mM)
Fig. 3

## Slide 4
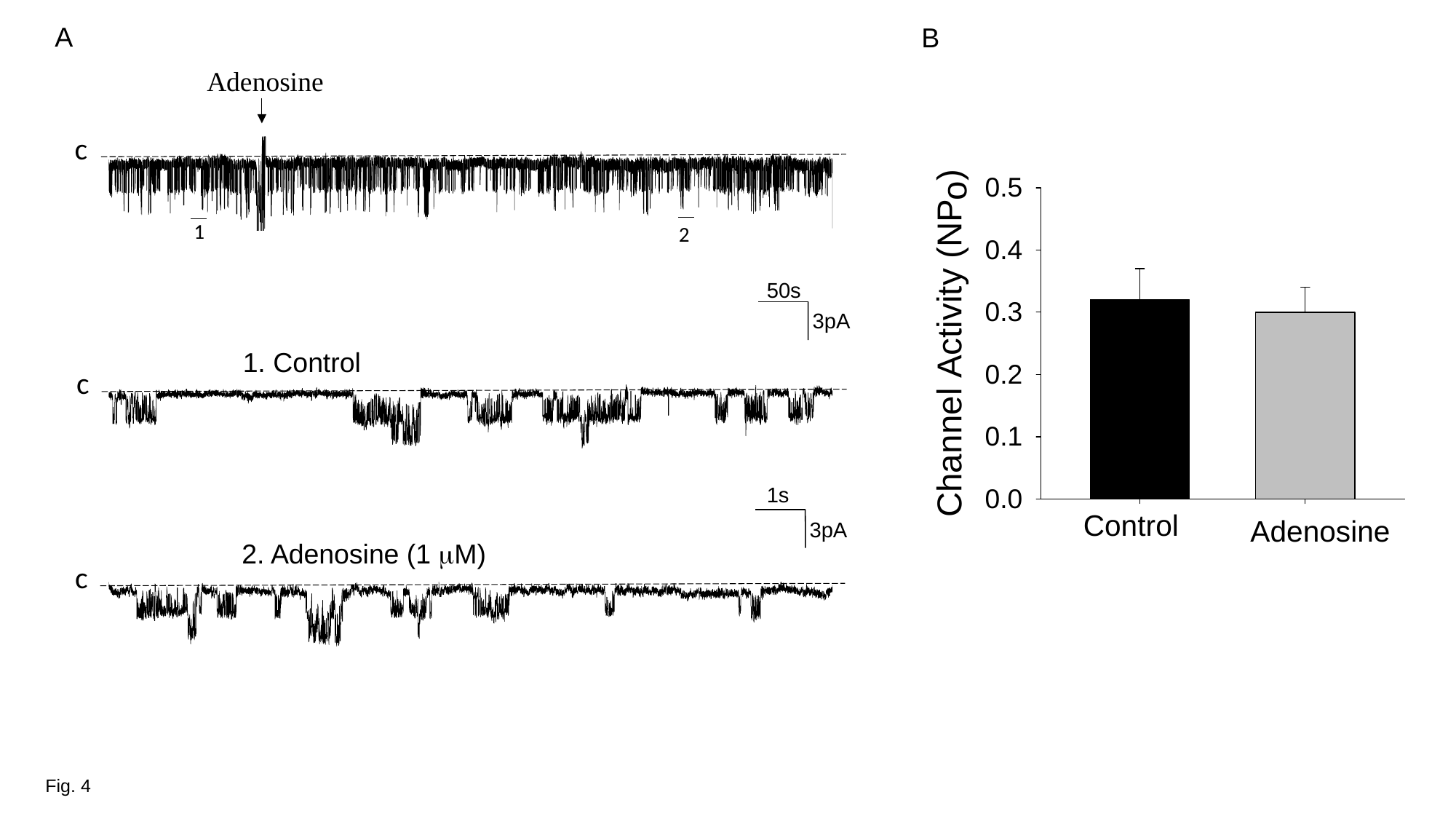

A
B
Adenosine
c
1
2
50s
3pA
c
1s
3pA
c
1. Control
2. Adenosine (1 mM)
Fig. 4

## Slide 5
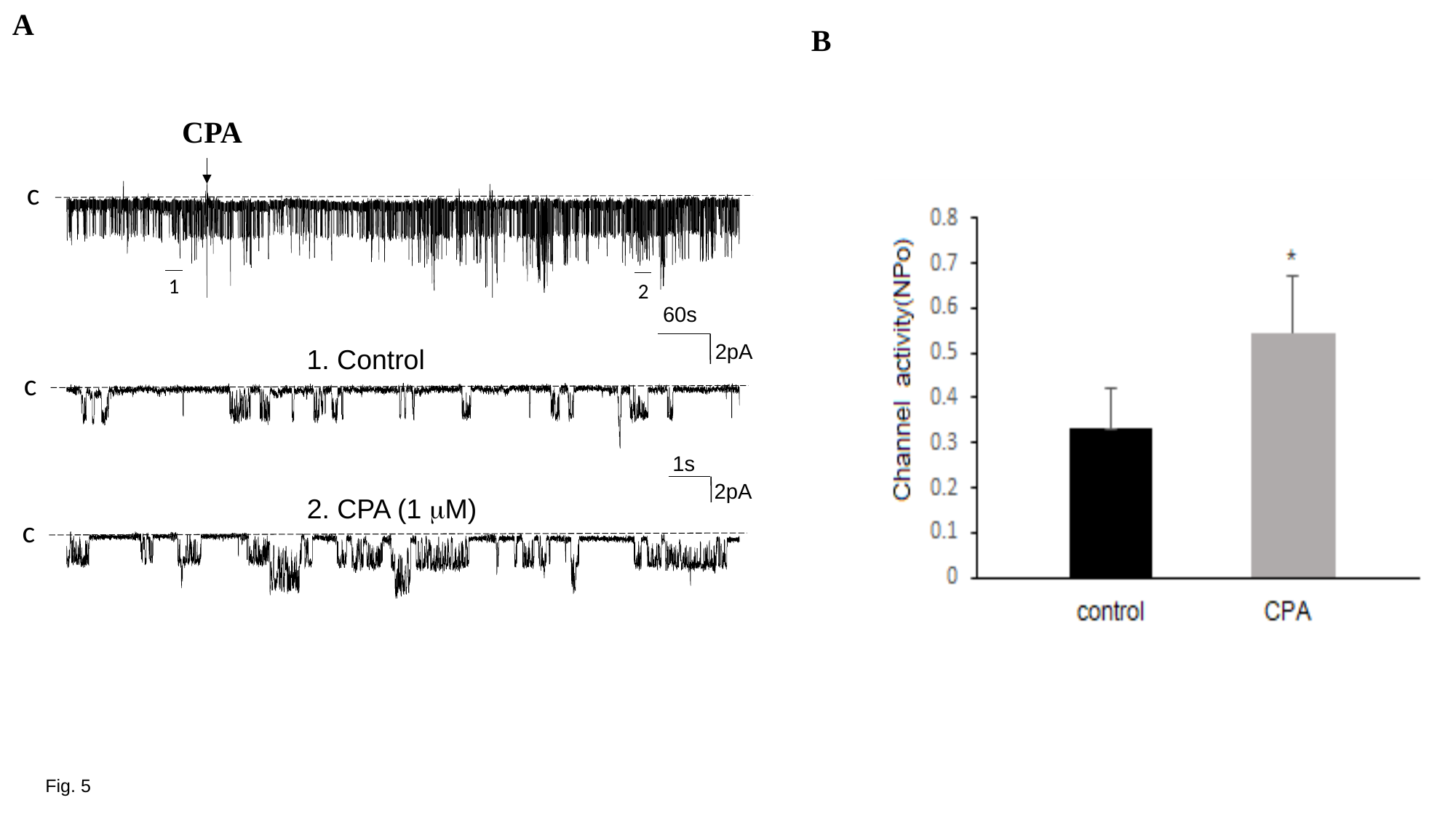

A
 B
 CPA
c
1
2
60s
2pA
c
1s
2pA
c
1. Control
2. CPA (1 mM)
Fig. 5

## Slide 6
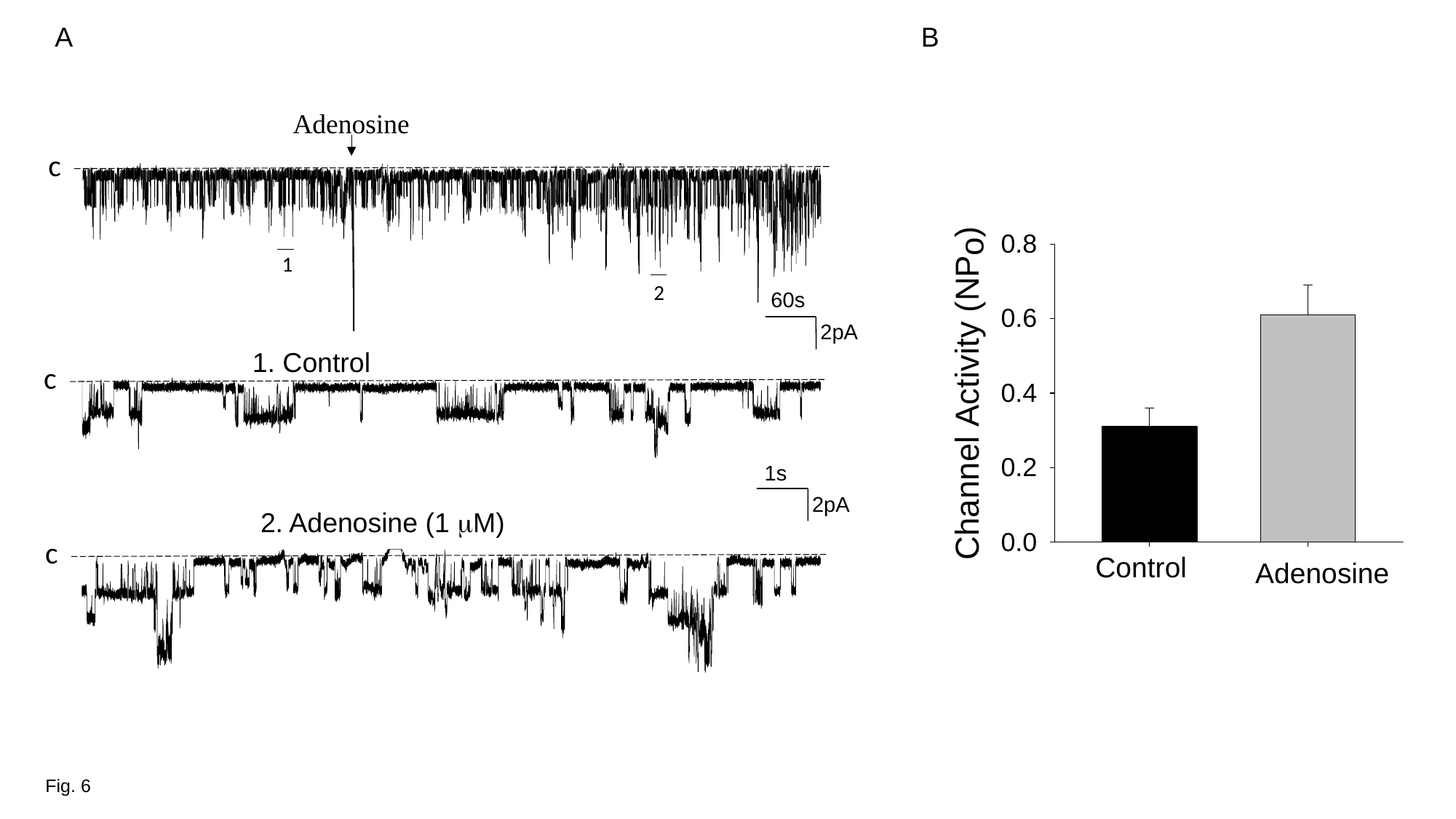

A
B
Adenosine
c
1
2
60s
2pA
c
1s
2pA
c
1. Control
2. Adenosine (1 mM)
Fig. 6

## Slide 7
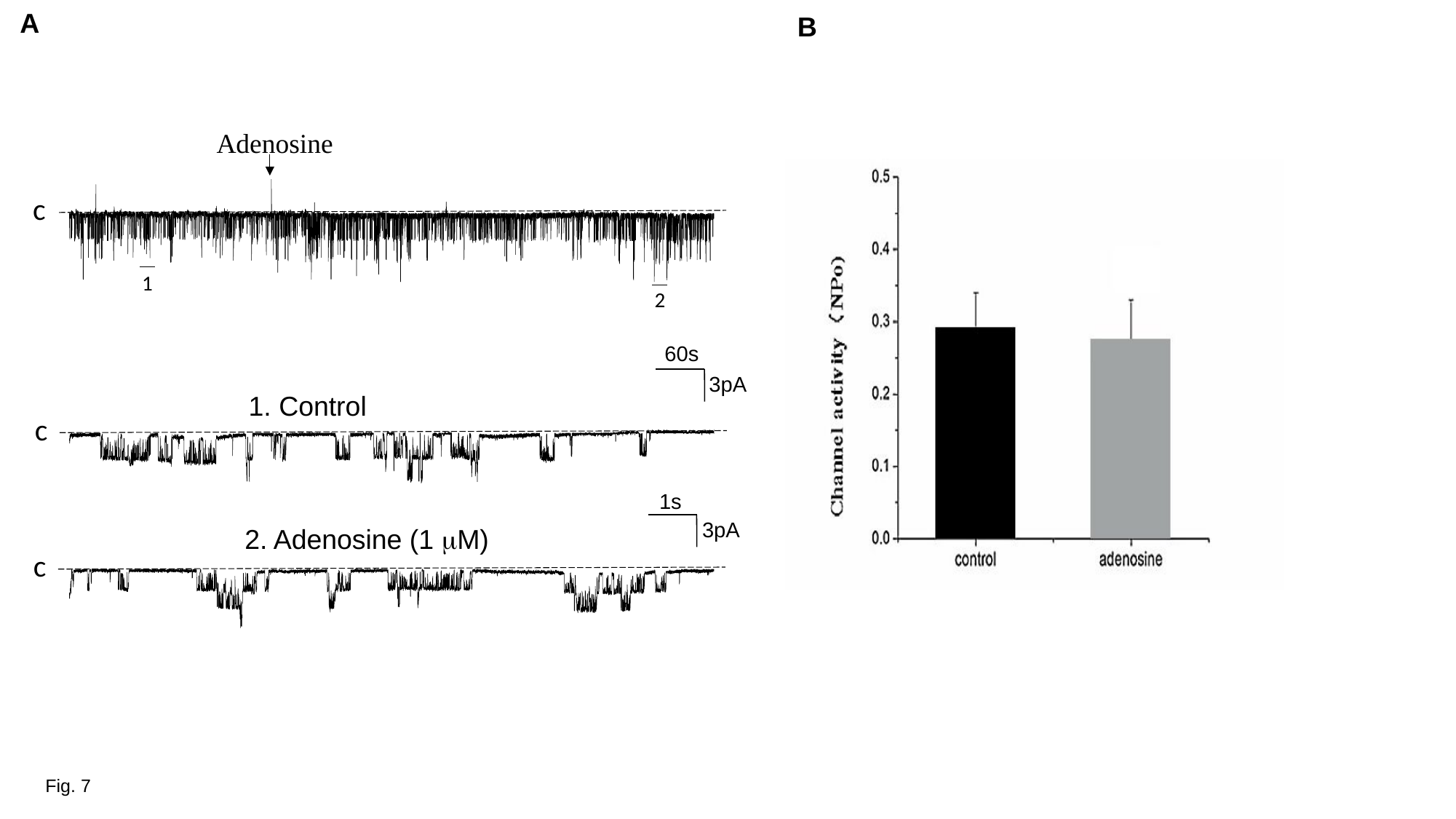

A
B
Adenosine
c
1
2
60s
3pA
c
1s
3pA
c
1. Control
2. Adenosine (1 mM)
Fig. 7

## Slide 8
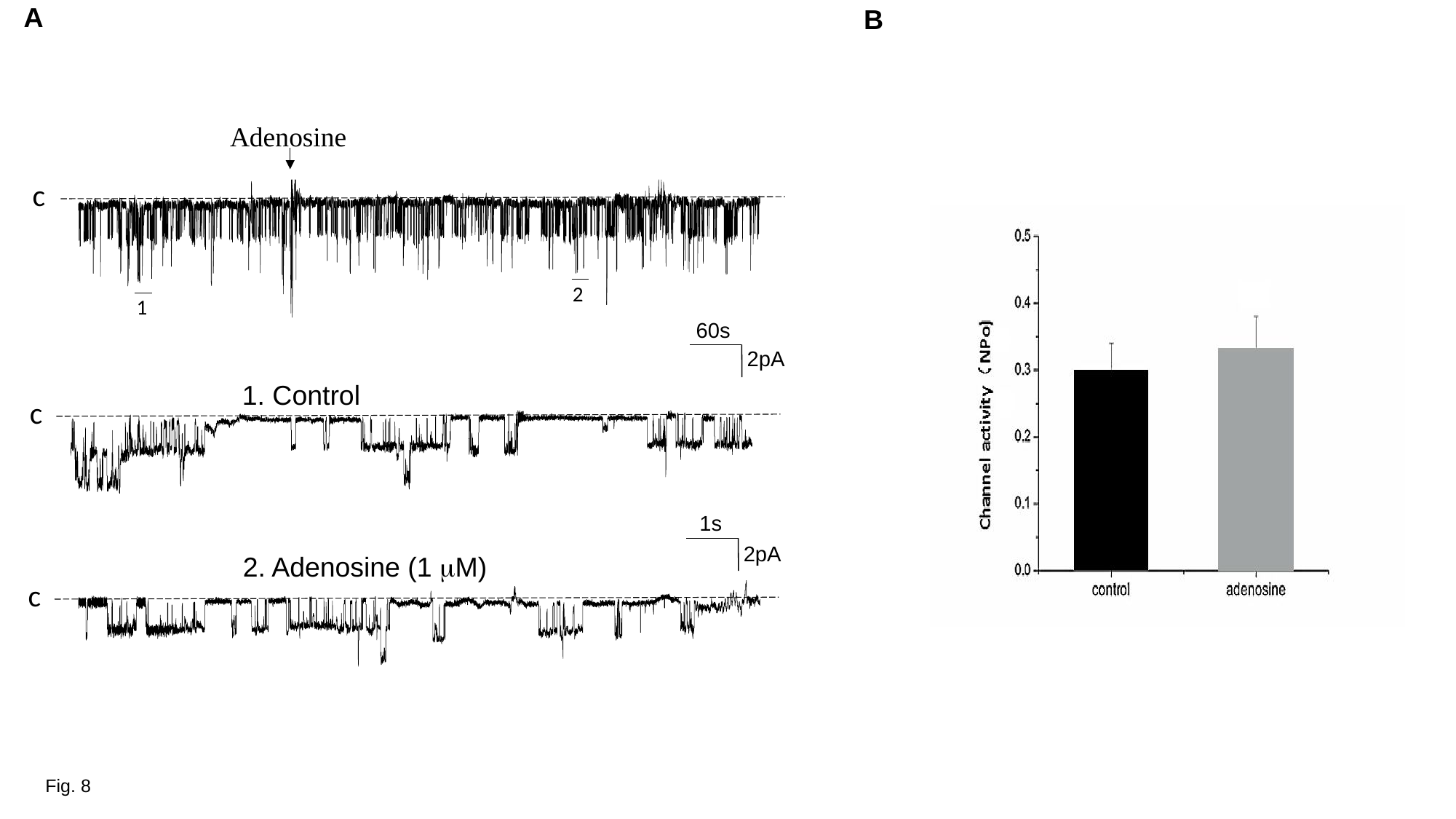

A
A
B
Adenosine
c
2
1
60s
2pA
c
1s
2pA
c
1. Control
2. Adenosine (1 mM)
Fig. 8

## Slide 9
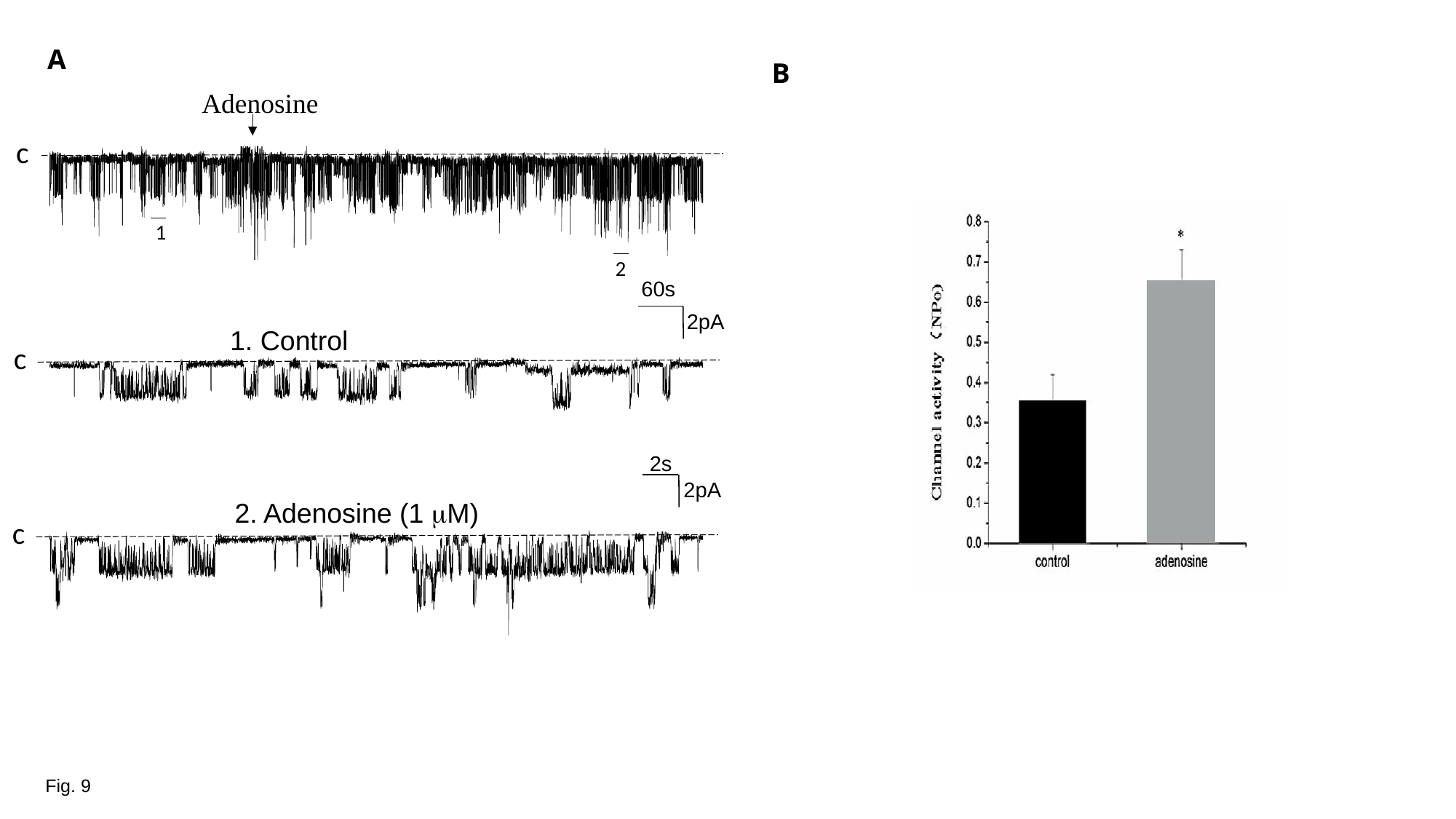

A
B
Adenosine
c
1
2
60s
2pA
2s
2pA
c
c
1. Control
2. Adenosine (1 mM)
Fig. 9

## Slide 10
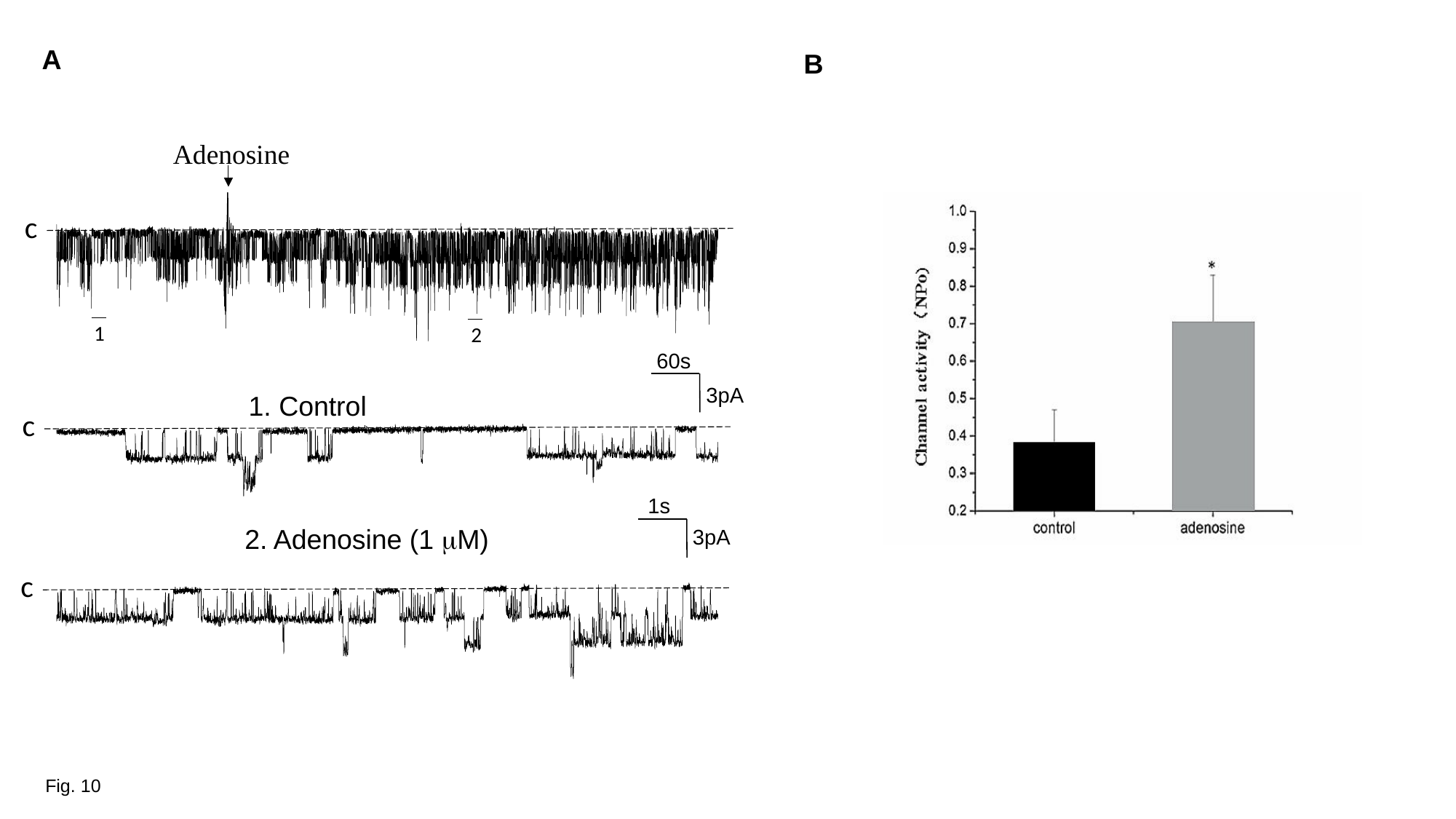

A
B
Adenosine
c
1
2
60s
3pA
c
1s
3pA
c
1. Control
2. Adenosine (1 mM)
Fig. 10

## Slide 11
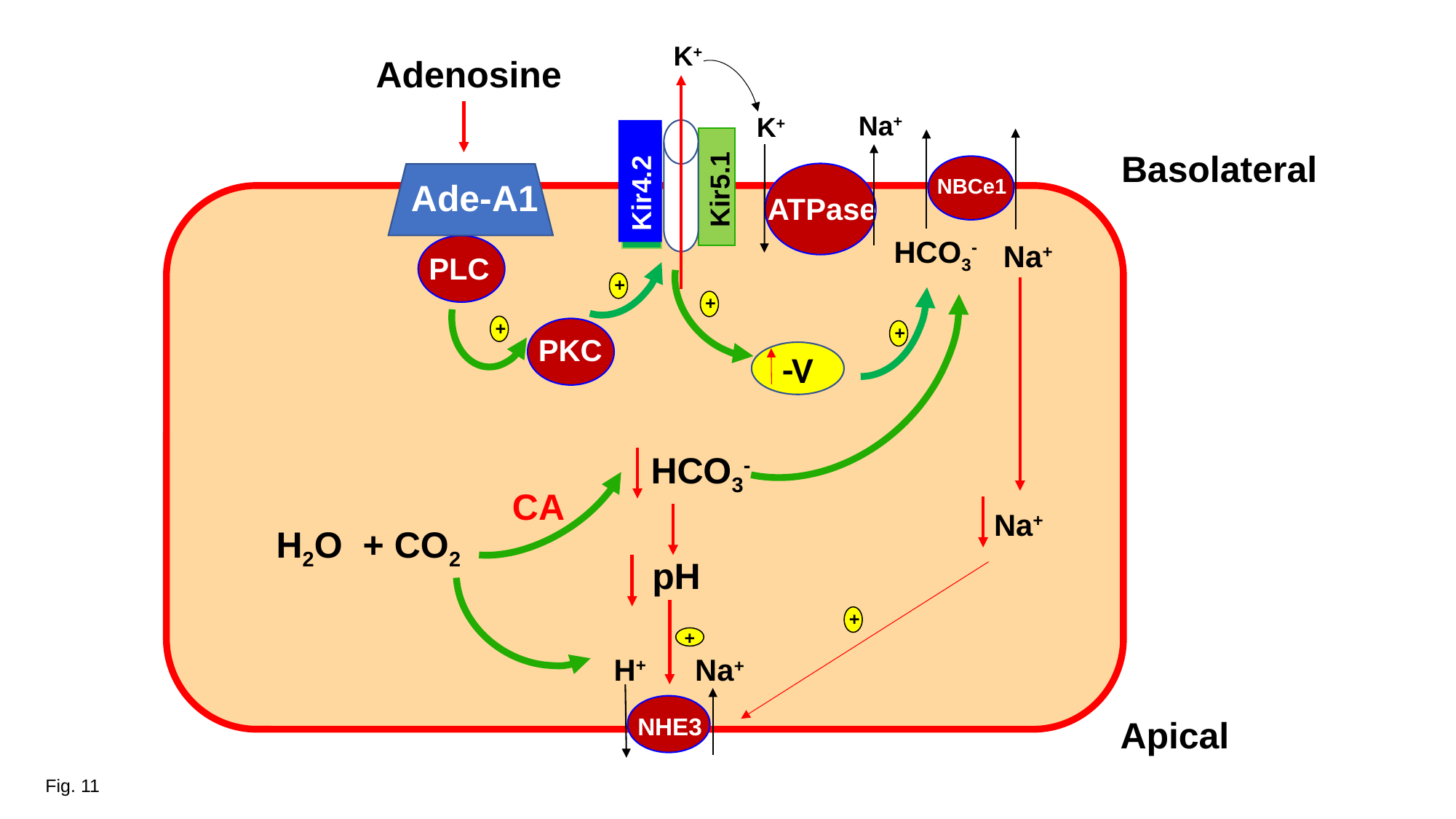

K+
Adenosine
Kir5.1
Kir4.2
Na+
K+
Basolateral
NBCe1
Ade-A1
ATPase
HCO3-
Na+
PLC
+
+
+
+
PKC
-V
HCO3-
CA
Na+
H2O + CO2
pH
+
+
H+
Na+
NHE3
Apical
Fig. 11
